# Seroepidemiology of Foot and Mouth Disease using passive surveillance techniques in selected provinces of Lao PDR

**DOI:** 10.1007/s11250-021-02734-y

**Published:** 2021-05-02

**Authors:** Jarunee Siengsanan-Lamont, Bounlom Douangngeun, Watthana Theppangna, Syseng Khounsy, Phouvong Phommachanh, Somjai Kamolsiripichaiporn, Romphruke Udon, Kingkarn Boonsuya Seeyo, Paul W. Selleck, Nina Matsumoto, Laurence J. Gleeson, Stuart D. Blacksell

**Affiliations:** 1 Maidol-Oxford Tropical Medicine Research Unit, Faculty of Tropical Medicine, Mahidol University, Bangkok, Thailand; 2 National Animal Health Laboratory, Department of Livestock and Fisheries, Ministry of Agriculture and Forestry, Vientiane, Lao People’s Democratic Republic; 3 Regional Reference Laboratory for Foot and Mouth Disease in South East Asia, Department of Livestock Development, Nakhon Ratchasima, Thailand; 4 Sydney School of Veterinary Science, University of Sydney, Camden, Australia; 5 Centre for Tropical Medicine & Global Health, Nuffield Department of Medicine, University of Oxford, Oxford, UK; 6 Lao-Oxford-Mahosot Hospital-Wellcome Trust Research Unit, Mahosot Hospital, Vientiane, Lao People’s Democratic Republic

**Keywords:** Foot and mouth disease, Laos, Seroepidemiology, Passive surveillance, Asia

## Abstract

Foot and Mouth Disease (FMD) is a high-impact, contagious transboundary animal disease that is endemic in Southeast Asia. Abattoir samples were routinely collected in six selected provinces between March and December 2019. A total of 1280 samples of abattoir animals were tested for FMD Non-Structural Protein (NSP) antibodies to indicate natural infections. Overall, 22.8% were seropositive for FMD NSP antibodies while seroprevalence of cattle (*n* = 469), buffalo (*n* = 214), and pigs (*n* = 597) were 44.6%, 35.0%, and 1.3%, respectively. The highest seroprevalence destination province was Xiengkhouang (35.3% of 272 samples), followed by Savannakhet (27.0% of 244 samples). Risk factors for evidence of natural infection identified by a multivariate logistic regression model included age groups (*p*-value = 0.02) and origin provinces (*p*-value = 2.8 × 10^−5^) of the animals. There were significant differences of FMD NSP seroprevalence between age groups and origin provinces of the animals. The odds ratio of a seropositive result in the less than 1 year old group was 2.5 (95% CI; 1.4, 4.4) when compared to the 3–4 years old group, while the odds ratios for animals that originated from Khammouane and Xiengkhouang provinces were 4.5 (95% CI; 1.1, 18.7) and 2.4 (95% CI; 1.4, 4.1), respectively, when compared to Champasak province. Serotype-specific antibody ELISA for 44 NSP antibody–positive samples revealed evidence of FMD serotypes O and A virus circulation in some provinces. Despite the passive abattoir survey providing useful information on FMD virus previous exposure and geographic locations of the animals, timely information on FMD virus circulation and distribution is also crucial to an effective control program. Alternative approaches to increase the cost-effectiveness of the surveillance network are also discussed.

## Introduction

Foot and Mouth Disease (FMD) is an important transboundary animal disease in Southeast Asia which results in significant economic impacts to countries in the region due to production losses and international trade disruption (OIE 2018). Lao PDR is an importer and transit country for transboundary cattle trade (Bounma 2019) and a member of the Southeast Asia and China Foot and Mouth Disease (SEACFMD) Campaign coordinated by the World Organisation for Animal Health (OIE 2020). In 2017, Lao PDR reported a livestock population (cattle, buffalos, pigs, and goats) of approximately seven million (Bounma 2019). Data collected between 2012 and 2016 in Southern Laos revealed a FMD seroprevalence of more than 50% in adult ruminants (older than 5 years) while an active survey in Xiengkhouang (XKG) province in 2017 demonstrated 33.2% seroprevalence (Bounma 2019). With the national objective to control FMD and promote livestock trade, information on FMD circulation and distribution, and a better understanding of the potential risk factors will contribute to a more effective disease control program.

The national animal health surveillance network initiated by the Department of Livestock and Fisheries (DLF) and National Animal Health Laboratory (NAHL) within the Ministry of Agriculture and Forestry has been developed under a collaboration with Mahidol Oxford Tropical Medicine Research Unit (MORU) and funded by the Defense Threat Reduction Agency (DTRA) of the U.S. government. The surveillance program aimed to utilize the existing network of the Provincial-level and District-level Agricultural and Forestry Offices (PAFO and DAFO) to collect animal disease information (including priority diseases incidence or outbreak data), and livestock samples for passive and active surveillance. Passive abattoir surveillance activities started in March 2019 and concluded in December 2020. The outcomes of the 2019 abattoir surveillance program specific to FMD are discussed in this paper. In the near future, once the network has been fully established, active surveillance in high-risk areas could be performed to provide better disease epidemiology.

## Materials and method

### Study design

The site selection and sample size calculation of the surveillance program were previously described in Siengsanan-Lamont et al. (2021). Targeted provinces Champasak (CPS), Luangnamtha (LNT), Luangprabang (LPB), Oudomxay (ODX), Savannakhet (SVK), and Xiengkhouang (XKG) represented the areas of higher livestock numbers and animal movements. Up to twenty cattle and/or buffalo and ten pigs were sampled twice a month at an abattoir in the six provinces, depending on the availability of abattoir livestock on the collection day. Blood samples and animal biodata were collected by trained PAFO and DAFO staff. Serum samples were separated at provincial laboratory facilities and stored at 4 °C until dispatched. The samples were then securely packed in an icebox (filled with ice or ice packs) and transported to the NAHL via bus or plane. Once the box arrived at the NAHL, the samples were stored at −20 °C and their biodata entered into the NAHL’s Pathogen Asset Control System (PACS).

### Laboratory testing

Serum samples were tested for antibodies against FMD 3ABC Non-Structural Protein (NSP) using the ID Screen® FMD NSP Competition ELISA (IDvet, France, Cat# FMDNSPC-10P). The ELISA was performed according to the manufacturer’s instructions provided in the kit, and the IDSoft^TM^ software (IDvet 2014) used to calculate the Sample to Positive Ratio (S/P%). Based on the manufacturer’s recommendations, samples with the S/P% < = 50% were considered positive, while the S/P% > 50% were negative. Results of the diagnostic tests were entered into the PACS for record-keeping. The test sensitivity and specificity were 91.7% and 99.5%, respectively (Roche et al. 2014). Seropositive swine samples and large ruminant (aged less than 1 year) samples were submitted to the Regional Reference Laboratory for Foot and Mouth Disease in South East Asia (RRL FMD), Pak Chong, Thailand. FMD serotype–specific antibody titers for serotypes O, A, and Asia1 were determined using the liquid phase blocking ELISA (LPBE) antibody titers specific to FMD Type O, A, and Asia1 equal or greater than 1:80 was considered positive.

### Data analyses

Microsoft Excel (Microsoft Corporation 2020) and R Studio Version 1.2.1335 (RStudio Team 2015) were used for descriptive statistical and spatial analyses. The test results and animal biodata (including age, body score, destination province, breed, sex, origin province, and species) were used to produce frequency and probability distributions. The apparent and true seroprevalences (Wilson method; Reiczigel et al. (2010)) were calculated. The leaflet R package (Graul 2016) was used to map animal movements for visualization. Multivariate logistic regressions using the test results as the dependent variable and biodata as independent variables were conducted to identify potential risk factors. Univariate logistic regression models were used to select a subset of variables (*p*-value < 0.1) for further multivariate analyses (Souriya et al. 2019). The variables in the subset were then tested for multicollinearity using the variance inflation factor (VIF) (DataCamp.com, 2020). Only one of the collinear variables (VIF > 10) (Curto and Pinto 2011) was kept in the multivariate logistic regression model. The backward stepwise selection was then performed to identify a potential final model. The final model had the lowest Akaine Information Criterion (AIC) compared to the other models generated in the stepwise selection. Analysis of variance (ANOVA) for the final model was calculated using chi-square (Fox 2020). Odds ratios (OR) and the 95% confidence intervals (CI) of the significant variables (*p* < 0.05) in the final model were derived from univariate logistic regression models (UCLA, Statistical Consulting Group 2020).

## Results

**Fig. 1:**
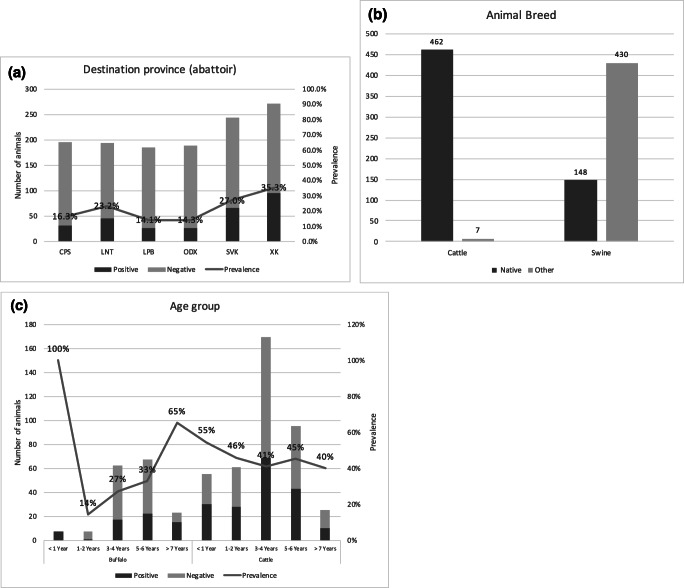
**a** FMD NSP seroprevalence relative to destination province. **b** FMD NSP seroprevalence relative to animal breed. **c** FMD NSP seroprevalence relative to age and species

A total of 1280 samples (597 swine, 469 cattle, and 214 buffalo) were collected from abattoirs in the six provinces of which 292 samples were positive in the FMD NSP Antibody ELISA. The overall apparent positive prevalence was 22.8% with 95% CI (20.6, 25.2) while the true prevalence was 24.5% (95% CI; 22.0, 27.1). XKG and SVN provinces had overall seroprevalences of 35.3% (*n* = 272) and 27.0% (*n* = 244), respectively following by LNT province of 23.2% (*n* = 194; Fig. 1).

### Large ruminants

**Fig. 2:**
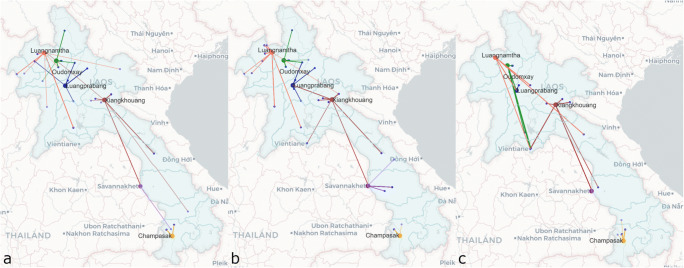
Animal movement maps (**a** buffalo; **b** cattle; **c** swine) from a source of origin (blue dot) to an abattoir in the six provinces (LNT =orange, ODX =green, LPB = Navy, XKG = brown, SVK = violet and CPS = yellow)

Seroprevalence of cattle and buffalo is presented in Table 1. By age, 59.6% (*n* = 62) of animals aged less than 1 year old and 52.1% (*n* = 48) of animals age more than 7 years old were seropositive to FMD NSP. More than 98% of cattle (*n* = 469) was recorded as a native breed. Animal movements comparing buffalo (Fig. 2a) and cattle (Fig. 2b) revealed similar movement trends across the species.

**Table 1:** FMD NSP seroprevalence by species

Species	Total	Positive	Apparent seroprevalence (95% CI)	True seroprevalence (95% CI)
Buffalo	214	75	35.0% (29.0, 41.7)	37.9% (31.2, 45.1)
Cattle	469	209	44.6% (40.1, 49.1)	48.3% (43.5, 53.3)
Swine	597	8	1.3% (0.7, 2.6)	0.9% (0.2, 2.3)
Total	1280	292	22.8% (20.6, 25.2)	24.5% (22.0, 27.1)

### Swine

Swine had the lowest FMD NSP seroprevalence (1.3%, *n* = 597) when compared to the other two species. Only eight swine samples were positive, of which four samples were collected in ODX (from exotic breed pigs at the same abattoir and collection date), three samples collected in XKG (2 native and one exotic breed), and one exotic breed sample in LNT (Table 2). The ages of swine were not recorded, although 69.0% of swine samples were indicated as exotic breed and 25.6% as native (*n* = 578). Both native (*n* = 148) and exotic breed (*n* = 430) swine had the same seroprevalence of 1.4%. Swine movement (Fig. 2c) was less extensive than the other two species.

**Table 2:** Overall FMD NSP seroprevalence comparing destination and origin provinces with species

% Seroprevalence (*n*)	Origin province	% Seroprevalence (*n*)		
Destination province (abattoir)		Buffalo	Cattle	Swine
Champasak (16.3%, 196)	Salavan	-	-	0% (1)
	Local	26.5% (68)	50% (28)	0% (99)
Luangnamtha (23.2%, 194)	Bokeo	50% (4)	50% (4)	-
	Bolikhamxay	-	-	0% (10)
	Houaphan	0% (1)	-	-
	Luangprabang	-	-	0% (36)
	Oudomxay	0% (1)	-	-
	Vientiane	60% (10)	50% (4)	0% (6)
	Xayabury	0% (1)	66.7% (9)	-
	Xiengkhouang	-	100% (1)	-
	Local	33.3% (15)	41.9% (43)	2.2% (46)
	Unknown	0% (1)	100% (2)	-
Luangprabang (14.1%, 185)	Local	36.7% (49)	22.2% (36)	0% (100)
Oudomxay (14.3%, 189)	Luangprabang	0% (3)	25% (8)	0% (34)
	Phongsaly	60% (5)	66.7% (3)	-
	Vientiane Prefecture	-	-	0% (18)
	Vientiane	-	-	0% (17)
	Local	8% (25)	28.6% (49)	0% (17)
	Unknown	-	-	40% (10)
Savannakhet (27.0%, 244)	Champasak	100% (1)	-	-
	Khammouane	-	0% (1)	-
	Local	0% (1)	45.5% (132)	0% (101)
	Unknown	0% (1)	71.4% (7)	-
Xiengkhouang (35.3%, 272)	Khammouane	75% (4)	80% (5)	-
	Luangprabang	-	33.3% (6)	-
	Salavan	0% (1)	-	-
	Savannakhet	66.7% (3)	66.7% (3)	5.6% (18)
	Vientiane Prefecture	-	-	0% (11)
	Vientiane	-	0% (2)	0% (19)
	Xaisomboun	0% (2)	80% (5)	-
	Local	83.3% (18)	50.4% (121)	3.7% (54)
Total		35.0% (214)	44.6% (469)	1.3% (597)

### Serological profiling of positive NSP samples

A total of 44 samples (36 large ruminants less than 1 year old and eight swine samples) were submitted to RRL FMD for further serological profiling. The profile results of the 36 ruminant samples showed 15 did not have detectable antibodies in the LPBE, 5 with antibodies to type A only, 5 with antibodies to type O only, 10 with antibodies to both A and O, and one with substantial titers to A, O, and Asia 1 (Table 3). Almost all positive LPBE ruminant samples were collected from XKG in October 2019 except one sample collected from LPB in November 2019. For the 8 swine samples, 2 had no detectable antibodies in the LPBE, 3 were positive to all three serotypes, one positive to A and O, one to A only, and one to A and Asia 1. Three LPBE-positive swine samples were collected from ODX (in May), two from XKG (in May and October), and one from LNT (in November).

**Table 3:** Summary of FMD LPBE-positive serology results

Sample collection date	Destination province (abattoir)	Origin province	Origin district	Age (months)	Animal breed	Species	FMD LPB ELISA titer (log10)		
							O	A	Asia1
29/03/2019	XKG	XKG	Paek	NA	Exotic	Swine	2.5	3.1	2.2
1/04/2019	ODX	NA	NA	NA	Exotic	Swine	2.8	3.1	2.5
		NA	NA	NA	Exotic	Swine	1.9	2.5	2.2
		NA	NA	NA	Exotic	Swine	1.6	2.2	1.9
11/07/2019	LNT	LNT	Namlue	NA	Exotic	Swine	1.6	1.9	1.6
24/10/2019	XKG	KHM	NA	4-6	Native	Cow	1.9	1.6	1.6
		KHM	NA	4-6	Native	Buffalo	1.6	1.9	1.6
		KHM	NA	4-6	Native	Cow	1.6	1.9	1.6
		SVK	NA	4-6	Native	Cow	2.5	3.1	1.6
		XSB	NA	4-6	Native	Cow	2.2	1.6	1.6
		XKG	Khoun	4-6	Native	Cow	1.6	1.9	1.6
		XKG	Khoun	4-6	Native	Cow	1.6	1.9	1.6
		XKG	Khoun	4-6	Native	Cow	2.2	1.6	1.6
		XKG	Paek	4-6	Native	Cow	2.2	2.2	1.6
		XKG	Paek	4-6	Native	Cow	2.2	2.5	1.6
		XKG	Paek	4-6	Native	Cow	2.2	2.8	1.6
		XKG	Paek	4-6	Native	Cow	2.2	2.2	1.6
		XKG	Paek	4-6	Native	Cow	1.9	1.6	1.6
		XKG	Paek	4-6	Native	Buffalo	2.2	1.6	1.6
		XKG	Paek	4-6	Native	Cow	1.9	1.9	1.6
		XKG	Phaxay	4-6	Native	Buffalo	1.6	2.2	1.6
		XKG	Phaxay	4-6	Native	Cow	3.1	3.4	2.2
		XKG	Phoukood	4-6	Native	Cow	2.5	3.1	1.6
		XKG	Phoukood	4-6	Native	Cow	1.6	1.9	1.6
		XKG	Phoukood	4-6	Native	Cow	2.5	2.8	1.6
		XKG	Phoukood	4-6	Native	Buffalo	2.2	1.9	1.6
		XKG	Nonghed	NA	Native	Swine	2.2	2.5	1.6
26/11/2019	LPB	LPB	Xieng Ngeun	5	Native	Cow	2.2	1.6	1.6
							2.1 (range: 1.6–3.1)	2.2 (range: 1.6–3.4)	1.7 (range: 1.6–2.5)

*CPS*, Champasak; *KHM*, Khammouane; *LNT*, Luangnamtha; *LPB*, Luangprabang; *ODX*, Oudomxay; *SVK*, Savannakhet; *XSB*, Xaisomboun; *XKG*, Xiengkhouang

### Risk factor analysis

Given that seroprevalence in swine was relatively low, only the buffalo and cattle data were included in the risk factor analyses to reduce potential bias. The variables with *p*-value <0.1, identified by univariate logistic regression analyses, were age group, breed, destination province, origin province, and species. The “destination province” variable (VIF = 2.5 × 10^11^) was excluded due to collinearity with the “origin province” variable. Only four variables were included in the multivariate logistic regression model using the backward stepwise selection. The final model (AIC = 908.25) had two significant independent variables including “age” (*p*-value = 0.02) and “origin province” (*p*-value = 2.8 × 10^-5^). Using the “3–4 years” age group as the reference group, the OR of the animal age less than the 1 year old group was 2.5 (95% CI; 1.4, 4.4) while the other age groups were not significant. The significant ORs of the origin province using “CPS” as the reference were Khammouane (KHM) (OR = 4.5, 95% CI; 1.1, 18.7) and XKG (OR = 2.4, 95% CI; 1.4, 4.1).

## Discussion

In this study, we have demonstrated the utility of passive surveillance techniques in six provinces of Lao PDR to gain a better understanding of the contemporary seroepidemiology of FMD. More abattoir samples were collected from XKG and SVK compared to the other four provinces, and most of the samples were from swine, followed by cattle and buffalo. A study of FMD NSP seroprevalence in pigs by Khounsy and Conlan (2008) reported a seroprevalence of 2.0% (*n* = 1563) in 2005 in four provinces and 2.9% (*n* = 947) in 2006 in five northern provinces. Another longitudinal abattoir survey conducted between 1999 and 2001 revealed that 18.7% (*n* = 9241; 27.4% of 1386 cattle, 32.1% of 2957 buffalos, and 8.1% of 4898 pigs) of sampled animals were seropositive against FMD viruses (Blacksell et al. 2008). Similar to Blacksell et al. (2008), our study revealed that the FMD seroprevalence in abattoir pigs was markedly less than that of buffalo and cattle, which reflects that most commercial piggeries have good control of FMD. The ID Screen® FMD NSP Competition ELISA kit detects antibodies against the 3ABC NSP which is highly conserved among the seven serotypes (O, A, Asia1, C, SAT1, SAT2, and SAT3) of FMD viruses (IDvet 2020). Anti-NSP antibodies generally indicated previous exposure to FMD viruses or recent virus infection (Mohanty et al. 2015). Our study used general estimates for the ID Vet NSP ELISA’s sensitivity and specificity to simplify the seroprevalence calculation. However, previous studies claimed the sensitivity and specificity of NSP ELISA kits varied (Fukai et al. 2018; IZS, Istituto Zooprofilattico Sperimentale 2004) by animal population, species, days after exposure and vaccination status, etc. Further studies to validate the ID VET ELISA kit using local animal samples will contribute to a better interpretation of the diagnostic results. Lesions or clinical signs in abattoir animals, which could help to confirm recent infection in combination with the positive serology, were not recorded in our study. When it is possible, information on animal health status, especially FMD specific lesions, should be noted in the abattoir surveillance sample collection form.

It should also be recognized that it has been previously demonstrated that vaccinated animals may develop antibodies against FMD NSP if vaccines contained traces of NSPs (Ma et al. 2011), and especially if animals have been vaccinated a number of times. One study also reported that the FMD NSP antibodies were detected by a 3ABC blocking ELISA kit for up to 3 years after exposure; however, the sensitivity of detecting previously infected animals reduced over time (Elnekave et al. 2015). One of the risk factors identified in our study (by the multivariate logistic regression model) was the age group. Given that FMD is an endemic disease in Southeast Asia (FAO 2019), it was likely that the older the animals are, the higher chance of having NSP antibodies either due to multiple vaccinations or previous exposure/infection(s). Cumulative FMD incidence data collected between 2012 and 2016 reported that FMD prevalence in calves (7–12 months) was 20% then increased to more than 50% in cattle older than 5 years (Bounma 2019). In our study, the seroprevalence of the less than 1 year old group was high compared to other age groups, and the odds of less than 1 year old animals tested seropositive was 2.5 times higher than the odds of seropositive animals age 3–4 years old. The 3–4 years old group represented the majority sampled and was used as the reference group. As animals in the less than one year old cohort were unlikely to be vaccinated multiple times, the antibody titer should represent relatively recent exposure to the FMD viruses, although it was some potential for maternally derived antibodies to be detected (Ferrari et al. 2016). In order to fully understand the underlying reason for the antibodies to NSP in this cohort, a selection of sera was sent to the reference laboratory for further serological investigation. In Lao PDR, FMD viruses serotype O predominately caused outbreaks while serotype A occurred sporadically (Blacksell et al. 2008; Blacksell et al. 2019). Recent outbreaks of serotype O were reported in late 2019 and early 2020 (Souriya 2020). An outbreak of serotype A was reported in 2018 (FAO 2019) in Attapeu province (Bounma 2019) while the last outbreak caused by serotype Asia1 in Lao PDR was officially reported in 1998 (WRLFMD 2020). In Southeast Asia, the last serotype Asia1 outbreak was reported in April 2017 in Myanmar (FAO, Food and Agriculture Organization of the United Nations 2019). However, a survey in 2011 determined that 3.6% (*n* = 615) of unvaccinated smallholder pigs tested positive to FMD Asia1 antibodies using solid-phase cELISA tests for FMD (Holt et al. 2019).

Serotyping results of the NSP seropositive swine samples and the less than 1 year old large ruminant samples suggested that some of these animals may have been previously vaccinated, received antibodies by transfer in colostrum, were exposed to natural infection, or a combination of these events. As animals are normally sold to abattoirs by traders, the vaccination history of these individual animals is unknown, and it was not feasible to attempt to search other project vaccination records for possible clues. LPBE-positive samples with antibodies against multiple serotypes, especially to all three serotypes, were most likely be antibodies induced by bivalent or trivalent vaccine. A study of FMD vaccine titers in cows revealed that LPBE titers peaked to approximately 2.5 (log10) around 20 days after the first vaccination (Puentes et al. 2016). Another study reported that vaccinated calves challenged with a natural aerosol of FMD virus resulted in LPBE antibody titers up to 3.2 compared to the mean titer up to 4.2 of control non-vaccinated calves (Hamblin et al. 1987). Thus, LPBE results revealed evidence of FMD serotype O and A circulation in the areas which is similar to a recent study by Xaydalasouk et al. (2020). However, our study cannot clarify that the antibodies were solely from natural infection. Further studies of duration of maternally derived FMD NSP antibodies in young animals and antibody serotyping using surveillance samples with known vaccination history would provide useful information. Additionally, while high titers (1:640 and 1:1280) indicated that the antigens used in the LPBE were matched to the field virus or vaccine, perhaps this was not the case for some NSP positive samples that did not react in the LPBE. An alternative explanation was that colostrum-derived NSP antibodies were higher in concentration than the serotype-specific antibodies in some young animals.

Although FMD is an OIE notifiable disease, there was no FMD event officially reported from Lao PDR to the World Animal Health Information Database (WAHIS Interface) for the past 10 years, with the last FMD event reported in 2008 (OIE, 2013). FMD incidences and outbreaks in Lao PDR were presented at the SEACFMD annual meetings and presentations were available online (OIE 2020). The incidence reports often related to specific areas or locations of interest, which may not be representative of the Laos livestock population. Our study collected samples from animals at slaughter provided useful information on the previous exposure to the FMD virus and geographical locations of the exposed animals; however, the survey outcomes do not represent the FMD seroprevalence of the entire animal population. In Lao PDR, FMD control measures included FMD vaccination in hotspot areas, advocacy for application of biosecurity, outbreak investigations, and emergency response, including movement control (Bounma 2019).

In 2012, mass vaccination of 100,000 livestock (buffalo and cattle) was conducted in XKG province together with a vaccine efficacy study organized by the OIE and Japan Trust Fund (JTF) (Sakamoto et al. 2016). Between 2012 and 2016, Lao PDR received up to 1,620,000 doses of FMD vaccine from the OIE Stop Transboundary Animal Diseases and Zoonoses (STANDZ) program funded from Australia and JTF. This vaccine was extensively used in high-risk areas in the Northern Laos (Nampanya et al. 2018). Since 2016, a pilot study of an FMD control program in Lao PDR funded by New Zealand Ministry of Foreign Affairs and Trade (MFAT) in collaboration with DLF, Lao PDR, and OIE provided some vaccination to livestock in the targeted areas in CPS, SVK, and XKG (McFadden et al. 2019). Bounma (2019) reported that between May and September 2018, more than 71,000 animals were vaccinated in CPS and SVK provinces. There was no locally produced FMD vaccine in the country and vaccination campaigns using mostly European manufactured vaccines matched to current circulating strains were promoted by international projects. Vaccine coverage and efficacy should be considered when implementing a vaccination program (Blacksell et al. 2019). The most common targeted vaccination coverage within herds was 80% and above, but as mentioned earlier, most vaccination was strategic and carried out along some of the trade routes to reduce outbreaks in higher risk resident populations. Even in these programs, however, the disease reproductive rate and proportion of animals with a protective level of specific antibodies should also be used to calculate appropriate coverage (Ferrari et al. 2016).

Livestock censuses reported a high density of cattle in XKG, SVK, and Vientiane and buffalo in SVK and CPS (Bounma 2019; Epprecht et al. 2018; Sisouphanthong and Taillard 2000). For the past 5 years, FMD outbreaks were reported predominately in the Northern area of Lao PDR (Souriya 2020). Areas in northern Laos considered FMD risk hotspots (Nampanya et al. 2018; Smith et al. 2015) included XKG, LNT, and Bokeo provinces, known as transit routes for transboundary animal movement (Bounma 2019). However, recent FMD outbreaks in 2019 and 2020 were reported in Oudomxay (northern) and Bolikhamxay (central) (Souriya 2020). Other identified FMD risk hotspots in central Laos were KHM (transit) and Vientiane capital (high consumption) and in southern Laos was SVK (high density and transit) (Bounma 2019). Our data revealed that the origin province where cattle and buffalo were raised was a risk factor for NSP seropositivity. The odds that seropositive animals originated from KHM and XKG provinces were 4.2 and 2.4 times higher respectively than the odds that seropositive animals originated from CPS province. The movements (Fig. 2) showed that XKG and LNT received livestock from more sources and over longer distances than other provinces. Despite the high density of cattle production in XKG, the movement map indicated a high demand for livestock in the area and hence high trading activity, perhaps driven by some transboundary movements to the markets in Viet Nam. Similar to our study, Bounma (2019) reported that generally cattle and buffalo for local consumption in XKG and other northern provinces were traded from south to north. On the other hand, abattoirs in SVK (south) sourced mainly local animals and some from neighboring provinces. The limited swine movement routes, as in Fig. 2c, may be a result of the main suppliers of pigs being more likely larger commercial farms rather than smallholders. Based on our data, approximately 65% were non-native breed. Again, all positive swine samples (except the unknown source of origin samples) originated from LNT, SVK, and XKG. The FMD serological profiles in some pigs suggested trivalent or bivalent vaccination, but without age data it was not possible to interpret the results further, in particular the presence of antibodies to NSP. Overall, the data from the pig surveillance suggests that the disease is relatively well controlled or does not effectively spill over in this population, as found in earlier studies in Thailand (Cleland et al. 1995).

The extensive animal movement in the country highlights a need for an effective monitoring system for not only FMD but also for other zoonotic, transboundary, and high-impact animal diseases, to provide timely disease distribution information and early detection of disease events. In January 2020, the national animal health surveillance network has been extended to cover all eighteen provinces. Since then, the network has collected abattoir samples regularly in the same manner as the 2019 activities discussed in this paper. A major challenge facing the long-term sustainability of the network is the limited human and financial resources. Central staff, PAFO, and DAFO are well trained and competent; however, staff numbers are limited, especially at the central veterinary laboratory. Sufficient funding to supply consumables (laboratory and field) and to provide financial support for field work is also critical. Alternative approaches to maximize cost-effectiveness and reduce expenses which will increase the sustainability of the network should be applied, for example using syndromic surveillance with sample collection in targeted areas, choice of diagnostic tests, and possible use of surveillance involving data submission by mobile phones.
